# Childhood diarrhea in high and low hotspot districts of Amhara Region, northwest Ethiopia: a multilevel modeling

**DOI:** 10.1186/s41043-016-0052-2

**Published:** 2016-05-16

**Authors:** Muluken Azage, Abera Kumie, Alemayehu Worku, Amvrossios C. Bagtzoglou

**Affiliations:** 1 Ethiopian Institute of Water Resources, Addis Ababa University, Addis Ababa, Ethiopia; 2 School of Public Health, College of Health Sciences, Addis Ababa University, Addis Ababa, Ethiopia; 3 Department of Civil and Environmental Engineering, School of Engineering, University of Connecticut, Storrs, CT 06269-3037 USA

**Keywords:** Childhood diarrhea, Children, Water, Sanitation, Multilevel analysis, Ethiopia

## Abstract

**Background:**

Childhood diarrhea is one of the major public health problems in Ethiopia. Multiple factors at different levels contribute to the occurrence of childhood diarrhea. The objective of the study was to identify the factors affecting childhood diarrhea at individual and community level.

**Methods:**

A cross-sectional study design was employed from February to March 2015 in high and low hotspot districts of Awi and West and East Gojjam zones in Amhara Region, northwest Ethiopia. Districts with high and low hotspots with childhood diarrhea were identified using SaTScan spatial statistical analysis. A total of 2495 households from ten (five high and five low hotspot) randomly selected districts were included in the study. A semi-structured questionnaire was used to collect data. Data were entered and cleaned in Epi Info 3.5.2 version and analyzed using Stata version 12. A multilevel logistic regression was used to identify factors associated with childhood diarrhea.

**Results:**

The prevalence of childhood diarrhea was 13.5 % and did not show significant variation between high [14.3 % (95 % CI 12.3–16.2 %)] and low [12.7 % (95 % CI 10.9–14.6 %)] hotspot districts. Individual- and community-level factors accounted for 35 % of childhood diarrhea variation across the communities in the full model. Age of children (6–35 months), complementary feeding initiation below 6 months, inadequate hand washing practices, limited knowledge of mothers on diarrhea, lowest wealth status of households, and longer time interval to visit households by health extension workers were factors for increasing the odds of childhood diarrhea at the individual level. At the community level, lack of improved water supply and sanitation and unvaccinated children with measles and rotavirus vaccine were the factors associated with childhood diarrhea.

**Conclusions:**

In this study, childhood diarrhea occurrences remained high. Both individual- and community-level factors determined the occurrence of diarrhea. Interventions should consider both individual- and community-level factors to reduce the occurrence of childhood diarrhea.

**Electronic supplementary material:**

The online version of this article (doi:10.1186/s41043-016-0052-2) contains supplementary material, which is available to authorized users.

## Background

Diarrhea remains one of the most common infectious diseases of children [1, 2]. The World Health Organization (WHO) estimated that globally, approximately 1.7 billion cases of childhood diarrhea occur each year [3]. Of the leading infectious causes of death worldwide, diarrhea was the second responsible for 578,000 deaths among children under 5 years of age in 2013 [2]. The burden of diarrheal diseases in developing countries is higher than developed countries [1, 4]. The greatest proportions of severe episodes of diarrhea occurred in the southeast Asian (26 %) and African regions (26 %) in 2010 [1].

In Sub-Saharan Africa, diarrhea accounted for 25 to 75 % childhood morbidity and 50 % childhood mortality [1]. In this region, rotavirus contributed the highest child death rate and remained the main cause of diarrhea [5]. In East Africa, the prevalence of childhood diarrhea was found in the range of 13–32 % [6–10]. Studies in different parts of Ethiopia showed that the prevalence of childhood diarrhea was in the range of 15–29 % [11–16]. However, the 2-week prevalence of childhood diarrhea at national level has shown a decline from 24 % in 2000 to 13.5 % in 2011 [6, 7].

Multiple factors contribute to the occurrence of diarrhea among children under 5 years of age. Childhood diarrhea was associated with low maternal education [8, 13, 15], age of children [8, 14–17], number of under five children [15, 17], latrine availability [15, 18], improper child stool disposal methods [15], mothers not practicing hand washing at critical times [13], lack of improved water sources [18–20], improper handling of drinking water [12, 16, 20], and improper refuse disposal [13, 14]. Systematic studies indicated diarrheal diseases, which are widespread in areas with water scarcity, unsafe drinking water supply, poor hygiene, and lack of sanitation which are poorly accessed [21, 22]. However, in rotavirus-vaccinated children, occurrence of childhood diarrhea decreased significantly [23–29].

In Ethiopia, including Amhara Region, provision of water supply and improvement in sanitation and hygiene have shown a progress in the past 10 years. According to WHO, the availability of improved drinking water supply in Ethiopia increased from 13 % in 1990 to 57 % in 2015 [30]. Improved and shared latrine facility availability in Amhara Region raised from 2 % in 2000 to 46 % in 2012 [31]. The promotion of hygiene and sanitation through the health extension program has been active since 2003 [32]. Rotavirus vaccine was launched in Ethiopia at the end of 2013 [33]. However, a facility-based report in Amhara Region showed that morbidity of childhood diarrhea was one of the top five leading cause of childhood morbidity in the past decade [34]. There is no recent information about childhood diarrhea at community level after implementation of the abovementioned interventions, particularly in the study area. Moreover, studies conducted in Ethiopia identified the determinants of childhood diarrhea using a standard logistic regression model, which has less power or increased type one error. Analyzing all factors at one level is likely to present a very incomplete picture on the evaluation of determinants of childhood diarrhea. The standard regression model assumes the presence of random variation between households, while neglecting the non-random variation of communities at different levels. An appropriate methodology is required for a more comprehensive and sound analysis.

Thus, a multilevel regression model, which controls the nesting effect of clusters at different levels, was used to account for the shortcomings of a standard logistic regression. The method was chosen for two reasons: First, it systematically analyzes the explanatory variables (covariate) at various levels of hierarchies that affect the outcome variable, or this model measures the interactions among covariates at different levels that affect the outcome variable. Second, it corrects the biases in parameter estimates resulting from clustering and provides correct standard errors [35]. Therefore, the aim of this study was to identify factors affecting childhood diarrhea at the individual and community level using multilevel regression analysis.

## Methods

### Study design and period

A cross-sectional study design was employed from February to March 2015 to assess the effect of individual- and community-level factors on childhood diarrhea in high and low hotspot districts.

### Study area

**Fig. 1:**
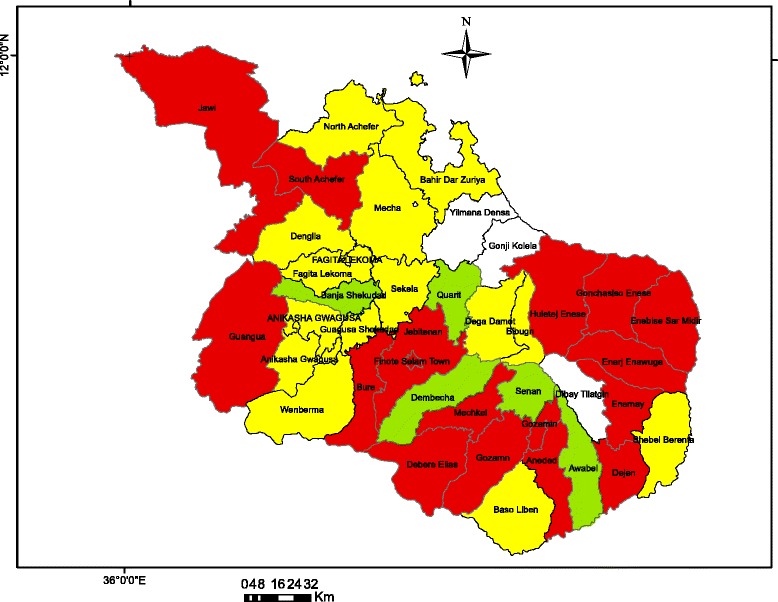
Map of study area (*red color* indicates districts with significant high hotspot of childhood diarrhea, *yellow color* indicates districts with significant low hotspot of childhood diarrhea, and *light green color* indicates districts with non-significant childhood diarrhea)

The study area included districts with high and low hotspots of childhood diarrhea in three (Awi and East and West Gojjam) zones in Amhara Regional State, northwest Ethiopia. Districts with high and low hotspots of childhood diarrhea were identified using SaTScan spatial statistical analysis. Of the 33 districts, 12 clusters, which covered 15 districts, were identified as a high hotspot of childhood diarrhea [36]. Eleven clusters, which encompassed 13 districts, were identified as a low hotspot of childhood diarrhea using the same data analysis procedure and these results are attached as supplement file (Additional file 1). The remaining five districts were non-significant districts (neither high nor low) in terms of childhood diarrhea spatial heterogeneity (Fig. 1).

### Study population, sample size, and technique

All children under 5 years of age in randomly selected districts were the study population. Children who lived in the study area for at least 6 months were used as inclusion criteria. The sample size was determined using Epi Info version 3.5.2 statistical software considering the following assumptions: 95 % confidence level, 80 % power with 1:1 ratio of diarrhea in high and low hotspots districts, 1.5 odds ratio, and 18 % prevalence of childhood diarrhea in low hotspot districts [11]. The calculated sample size was 1208. By considering a design effect of 2 and 5 % non-response rate, the final sample size was 2544 (1272 in low hotspot and 1272 in high hotspot districts).

A multistage random sampling technique was implemented to select the study population. At the first stage, ten districts (five low and five high hotspot districts) were selected randomly. At the second stage, one urban and one rural kebele from each low and high hotspot randomly selected districts were chosen using simple random sampling technique. Proportional to size allocation was made for each district and each kebele within a district. Households from randomly selected urban and rural kebeles were chosen using a systematic random sampling technique. The total number of households in each kebele was divided by the allocated sample size to get the sampling interval. Mothers of the under-fives were the respondents in a household. If there were more than one mother with children under 5 years of age in the same household, one mother was selected by lottery method. If there was no child in the identified household, the next household was used as sampling unit.

### Data collection tools

A structured questionnaire and observational checklist were used to collect data. The questionnaire was developed after reviewing related literatures and attached as supplement file (Additional file 2). The questionnaire includes variables on socio-demographic and economic data, knowledge of mothers/care takers on causes, transmission and prevention methods of childhood diarrhea, drinking water source and environmental sanitation, and childhood diarrhea in the past 2 weeks. Observational checklist was used to observe water storage container, the presence or absence of kitchen, availability and type of latrine, and presence or absence of hand washing facilities. The questionnaire was pre-tested in a similar setting after translating into the local language, Amharic. Data collectors and supervisors were recruited and then trained on the objective of the study and methods of the survey.

### Measurement of outcome variable

The prevalence of childhood diarrhea was measured using the WHO-recommended definition, namely if a child had three or more loose stools or watery diarrhea in a day during the 2 weeks preceding the study [37].

### Explanatory variables

Individual- and community-level variables that affect childhood diarrhea as described in Table 1 were included in the study. Coding for each explanatory variable and definitions of some of variables were also stated in the same table.

**Table 1:** Definitions and measurement of variables included in the models, northwest Ethiopia, 2015

Variables	Measurement of variables
Outcome variable	
Presence of childhood diarrhea	Categorized into (1) yes or (0) no
Individual-level factors (level one)	
Child factors	
Age of child (months)	Categorized into (1) 0–5, (2) 6–11; (3) 12–23; (4) 24–35; (5) 36–47; or (6) 48–59
Sex of child	Categorized into (1) female or (2) male
Complementary feeding at	Categorized into (1) ≥6 months or (2) <6 months
Parents/household factors	
Relation of child	Categorized into (1) mother; (2) care giver
Maternal age in years	Categorized into (1) 20–29; (2) 30–39; (3) 40–49 or (4) ≥50
Educational level of mother	Categorized into (1) no formal education; (2) primary; (3) secondary; or (4) higher
Occupation of mother	Categorized into (1) not working; (2) daily laborer; (3) farmer; or (4) employee or (5) merchant
Number of children	Categorized into (1) 1; (2) ≥2
Family size	Categorized into (1) ≤5; (2) >5
Comprehensive knowledge on diarrhea	Categorized into (1) knowledgeable; (2) limited knowledge
Methods of water drawing	Categorized into (1) deeping; (2) pouring
Hand washing practice of six critical times	Categorized into (1) practicing three and above critical times; (2) fail to practice at least three critical times
Presence of hand washing facility with water	Categorized into (1) yes; (2) no
Frequency of visiting of health extension workers	Categorized into (1) once in every 3 months; (2) once in every 6 months; (3) once above 6 months
Wealth index	Categorized into (1) poor; (2) medium; (3) rich
Community-level factors (level two)	
Residence	Categorized into (1) rural or (2) urban
Spatial heterogeneity	Categorized into (1) significant high number of childhood diarrhea (high hotspot) or (2) significant lower number of childhood diarrhea (low hotspot)
Refuse disposal	Categorized into (1) proper (if solid wastes are burying in private or communal pit or collected by municipal service or burning); (0) improper (open fields)
Drinking water sources	Categorized into (1) improved; (2) unimproved
Latrine facility type	Categorized into (1) improved; (2) unimproved, (3) unavailable
Rota vaccine status	Categorized into (1) vaccinated or (0) not vaccinated
Measles vaccine status	Categorized into (1) vaccinated or (0) not vaccinated

Comprehensive knowledge on diarrhea was categorized based on the mean score value of 13 items included in the questionnaire on the causes of diarrhea, means of transmission, and methods of prevention. These questions had “yes” or “no” responses, and a classification of “knowledgeable” was assigned if a respondent answered correctly seven or more items and “limited knowledge” if a respondent had six or less correct answers. Mothers were asked about their hand washing practices during critical times (after visiting a toilet, after cleaning a child’s bottom, after cleaning a house, before food preparation, before feeding a child, and before fetching water) and categorized based on mean score value, “practicing three or more hand washing critical times” and “practicing less than three hand washing critical times.”

Household wealth status was assessed as an indicator of socioeconomic status and was computed by principal component analysis from ten variables (presence of own farmland, own toilet facility, bank account, mobile phone, electricity, living house roofed with corrugated iron sheet, number of cows/oxen, horses/mules/donkeys, goats/sheep and chicken). Components resulting from the analysis were used to categorize households into three groups of wealth status (poor, medium, and rich) based on tercile.

Community-level factors were identified by global experts as indicators for evaluating diarrhea prevention activities at a given cluster or district (rotavirus and measles vaccine coverage, proportion of population with access to improved sanitation, proportion with access to improved drinking water); they were described in detail and published elsewhere [38].

### Data quality assurance

The questionnaire was pretested to evaluate the face validity and to ensure whether the study participants understood what the investigators intended to know. Training was given to data collectors and supervisors on how to select household and study participants and interviewing techniques. Daily supervision was made by the principal investigator to check the completeness of the questionnaire and consistency of related data. Data were coded, entered, and cleaned using Epi Info version 3.5.2 statistical software.

### Data analysis

Data were entered into Epi Info version 3.5.2 statistical software and exported to SPSS version 21. Data analysis was carried out using the statistical software Stata version 12. Descriptive statistics were used to describe the data. Multilevel logistic regression was used to analyze factors associated with childhood diarrhea at individual and community levels. Multilevel regression analysis used the scheme on a random model [39], considering two levels of data organization. Four models were constructed for doing this regression analysis. The first model, an empty model, was without any explanatory variable to evaluate the extent of cluster variation affecting childhood diarrhea. The second model controlled for the individual-level variables, the third model controlled for community-level variables, while the fourth model controlled for both the individual- and community-level variables simultaneously. Those variables with a *p* value of less than 0.2 in the second and third models were retained for the final model. The *p* value of <0.05 was used to define statistical significance. Adjusted odds ratio (AOR) with the corresponding 95 % confidence interval (CI) was calculated to identify factors affecting childhood diarrhea. Intra-cluster correlation (ICC), median odds ratio (MOR), and proportional change in variance (PCV) were calculated to measure the variation between clusters. ICC was used to explain cluster variation while MOR was used to measure unexplained cluster heterogeneity [40]. The ICC, MOR, and PCV formulae have been described elsewhere in detail [41–43].

### Ethical considerations

Ethical clearance was approved by the Research Ethics Committee of College of Medicine and Health Sciences, Bahir Dar University. The committee provided ethical approval after reviewing informed verbal consent submitted with all components of the research protocol. Permission letters from the Amhara Regional Health Bureau and district health offices were also obtained before starting data collection. The verbal consent was included on the front page of the questionnaire below briefing statements. Data collection was done after briefing the purpose of the study. Before data collection, study participants were asked for their willingness/unwillingness by marking their yes/no response. After confirming their verbal consent, interview and observation of the housing condition and environmental sanitation were conducted. Study participants were informed to interrupt the interview on desire. Confidentiality was insured by collecting the data anonymously, and the questionnaires were kept locked.

## Results

### Characteristics of study participants

Data were collected from 2544 households. Forty-nine (1.9 %) questionnaires were incomplete and were not included in the analysis. Of the completed data (2495), 1254 (50.3 %) and 1241 (49.7 %) were from high and low hotspot districts, respectively. Most participants in high (80.9 %) and low hotspot (85.7 %) districts lived in rural areas. The mean age of mothers was 29.5 (±6.1) in high and 29.6 (±6.7) in low hotspot districts. The overall mean age of mothers was 29.6 (±6.4) with a range of 20 and 70 years. The majority of mothers in high (51.1 %) and low (48.8 %) hotspot districts were found in the age range of 30–39 years. About 61.5 % of mothers in high and 71.8 % in low hotspot districts were unable to read and write. Nearly two third (64.4 %) of mothers in high and 79.1 % in low hotspot districts were farmers. Above 85 % of mothers in high (87.7 %) and low (93.4 %) hotspot districts were married and almost all mothers in both high (99 %) and low (98.7 %) hotspot areas were Orthodox Christian followers. Almost half of children in high (49.6 %) and 56.1 % in low hotspot districts were males. The mean age of children in high and low hotspot districts was 27.6 (±15.8) and 28.1 (±15.6) months, respectively. The overall mean age of children was 27.8 (±15.7) months. Nearly one fourth of children age in high and low hotspot areas were found in the range of 36–47 months. Almost equal percent of households in high (88.8 %) and low (89.2 %) hotspot districts had one child. Nearly one third of households in high (31.9 %) and 39 % in low hotspot districts had six and more family members. Thirty nine (34.9 %) of households in high and 25.9 % in low hotspot districts had a poor wealth index (Table 2).

**Table 2:** Socio-demographic and economic characteristics of households in high and low hotspot districts of Amhara Region, northwest Ethiopia, February–March 2015

Variables	HH in high hotspot districts		HH in low hotspot districts		*X* ^2^ test	*p* value
	No	%	No	%		
Place of residence						
Urban	239	19.1	177	14.3	10.32	0.001
Rural	1015	80.9	1064	85.7		
Relation of the mothers/care giver to the child						
Mother	1234	98.4	1210	97.5	2.54	0.111
Care giver	20	1.6	31	2.5		
Mothers/care givers age (years)						
20–29	175	15.2	170	14.4	8.03	0.045
30–39	589	51.1	576	48.8		
40–49	332	28.8	345	29.2		
≥50	56	4.9	90	7.6		
Mothers’ education						
Unable to read and write	771	61.5	891	71.8	51.584	<0.001
Able to read and write	77	6.1	95	7.7		
Primary	182	14.5	141	11.4		
Secondary	130	10.4	64	5.1		
Higher	94	7.5	50	4.0		
Occupation of mothers						
Not working	253	20.2	132	10.6	78.8578	<0.001
Daily laborer	68	5.4	51	4.1		
Farmer	808	64.4	982	79.1		
Employee	71	5.7	60	4.8		
Merchant	54	4.3	16	1.3		
Marital status of the mother						
Married	1100	87.7	1159	93.4	29.3206	<0.001
Single	13	1.0	3	0.2		
Widowed	21	1.7	21	1.8		
Separated	39	3.1	19	1.5		
Divorced	81	6.5	39	3.1		
Religion						
Orthodox Christian	1242	99.0	1220	98.7	2.58	0.108
Others	12	1.0	21	1.7		
Parents’ education						
Unable to read and write	380	30.3	468	37.7	62.69	<0.001
Able to read and write	258	20.6	248	28.0		
Primary	263	21.0	211	17.0		
Secondary	126	10.1	72	5.8		
Higher	125	10.0	82	6.6		
Occupation of parents						
Daily laborer	78	6.2	33	2.7	67.305	<0.001
Farmer	792	63.2	960	77.4		
Employee	202	16.1	149	12.0		
Merchant	80	6.4	39	3.1		
Child sex						
Male	622	49.6	696	56.1	10.5181	0.001
Female	632	50.4	545	43.9		
Child’s age (months)						
0–5	139	11.1	129	10.4	11.026	0.051
6–11	108	8.6	86	6.9		
12–23	263	21.0	236	19.0		
24–35	250	19.9	293	23.6		
36–47	287	22.9	260	21.0		
48–59	207	16.5	237	19.1		
Number of under five children						
One child	1114	88.8	1107	89.2	3.979	0.137
Two children	139	11.1	128	10.3		
Three children	1	0.1	6	0.5		
Family size						
≤5	854	68.1	757	61.0	13.76	<0.001
>5	400	31.9	484	39.0		
Wealth index						
Poor	438	34.9	313	25.2	50.48	<0.001
Medium	416	33.2	578	46.6		
Rich	400	31.9	350	28.2		

### Childhood diarrhea prevalence and related factors

The prevalence of childhood diarrhea was 14.3 % (95 % CI 12.3–16.2 %) in high and 12.7 % (95 % CI 10.9–14.6 %) in low hotspot districts. The overall prevalence of childhood diarrhea was 13.5 % (95 % CI 12.2–14.8%) in the study area. The proportion of children vaccinated with rotavirus vaccine was 63.3 % in high and 76.4 % in low hotspot districts, and the proportion of children vaccinated with measles vaccine were 82.0 % in high and 84.3 % in low hotspot districts. Children who started complementary feeding after 6 months were 79.7 and 77.4 % in high and low hotspot districts, respectively. About 35.0 % of mothers in high and 33.4 % in low hotspot districts had comprehensive knowledge on diarrhea. One third of mothers in high (32.9 %) and 30.4 % in low hotspot districts reported that they washed their hands at least three critical hand washing times (Table 3).

**Table 3:** Prevalence of childhood diarrhea and related factors among households in high and low hotspot districts of Amhara Region, northwest Ethiopia, February–March 2015

Variables	HH in high hotspot districts		HH in low hotspot districts		*X* ^2^ test	*p* value
	No	%	No	%		
Childhood diarrhea						
Yes	179	14.3	158	12.7	1.27	0.260
No	1075	85.7	1083	87.3		
Rota vaccine						
Yes	794	63.3	948	76.4	50.585	<0.001
No	460	36.7	293	23.6		
Measles vaccine						
Yes	1028	82.0	1046	84.3	2.37	0.124
No	226	18.0	195	15.7		
Complementary feeding at						
After 6 months	1000	79.7	961	77.4	7.988	0.018
Before 6 months	100	8.0	82	6.6		
Not remember	154	12.3	198	16.0		
Comprehensive knowledge on diarrhea						
Knowledgeable	495	39.5	414	33.4	0.7512	0.386
Limited knowledge	759	60.5	827	66.6		
Hand washing practice of six critical times						
Practicing at least three critical times	412	32.9	377	30.4	1.768	<0.001
Fail to practice at least three critical times	842	67.1	864	69.6		
Refuse disposal						
Proper	790	63.0	742	59.8	2.708	0.100
Improper	464	37.0	499	40.2		
Latrine type						
Improved	409	32.6	538	43.4	70.1315	<0.001
Unimproved	539	43.0	336	27.1		
Had no latrine	306	24.4	367	29.6		
Presence of hand washing facility with water						
Yes	82	6.5	38	3.1	16.47	<0.001
No	1172	93.5	1203	96.9		
Main source of drinking water						
Improved	770	61.4	809	65.2	3.8476	0.050
Unimproved	484	38.6	432	34.8		
Methods of water drawing						
Pouring	694	55.3	1095	88.2	332.595	<0.001
Deeping	560	44.7	146	11.8		
Presence of separated kitchen						
Yes	1069	85.3	795	64.1	149.181	<0.001
No	185	14.7	446	35.9		
Frequency of visiting of health extension workers						
Once in every 3 months	982	78.3	718	57.9	123.178	<0.001
Once in every 6 months	162	12.9	276	22.2		
Once above 6 months	110	8.8	247	19.9		

Above 60 % of households in high (63.0 %) and almost 60 % in low (59.8 %) hotspot districts used proper refuse disposal methods. Presence of latrine in the households was 75.6 % in high and 70.5 % in low hotspot districts, of which only 32.6 and 43.4 % had improved latrine facilities, respectively. Only 6.5 % of households in high and 3.1 % in low hotspot districts had hand washing container with water at a location nearby their latrine. About 61.2 % of households in high and 65.2 % in low hotspot districts used improved water for drinking and domestic purpose, and above half of households in high (55.3 %) and 88.2 % in low hotspot districts used pouring method to take water from a water storage container. More than 85 % of households in high and 64.1 % in low hotspot districts had separate kitchens for food preparation. Above three fourth of households in high (78.3 %) and 57.9 % in low hotspot districts had been visited by health extension workers once in every 3 months (Table 3).

### Multilevel analysis

The results of multilevel logistic regression models for individual- and community-level factors are displayed in Table 4. Child sex, mother’s relation to a child, maternal education and occupation, family size, numbers of children, water handling, presence of kitchen, and availability of hand washing facility in the second model and spatial heterogeneity, place of residence, and refuse disposal methods in the third model did not show statistically significant association with childhood diarrhea. Of all the factors included in the full model for multilevel analysis, the child’s age, complementary feeding initiation at 6 months, hand washing practice at least three critical hands washing times, comprehensive knowledge on childhood diarrhea, wealth index, frequency of visitation by health extension workers, drinking water source, latrine status, and measles and rotavirus vaccination status were significantly associated with childhood diarrhea. The odds of developing childhood diarrhea among children in the age group between 6–11 months, 12–23 months, and 24–35 months were 5.87 times (AOR 5.87; 95 % CI 3.00–11.46), 3.31 times (AOR 3.31; 95% CI 2.07–5.29), and 1.80 times (AOR 1.80; 95 % CI 1.11–2.92) higher than those children whose age was greater than 47 months, respectively. The odds of developing childhood diarrhea among those children who started complementary feeding below 6 months were 6.81 times (AOR 6.81; 95 %CI 4.52–10.25) higher than those children who started complementary feeding above 6 months. The odds of having childhood diarrhea among children whose mothers failed to practice at least three hand washing critical times were 1.70 times (AOR 1.70; 95 % CI 1.20–2.40) higher than those children whose mothers practiced at least three critical times. The odds of developing childhood diarrhea among children whose mothers had limited comprehensive knowledge on childhood diarrhea were 1.45 times (AOR 1.45; 95 % CI 1.06–1.97) higher than those children whose mother had comprehensive knowledge on childhood diarrhea. The odds of having diarrhea in children who were from low/poor household wealth index were 1.63 times (AOR 1.63; 95 % CI 1.12–2.36) higher than those who were from the rich household wealth index. The odds of having diarrhea in children whose house revisit interval by health extension workers was longer than 6 months were 1.7 times (AOR 1.72; 95 % CI 1.13–2.60) higher than in children whose houses were revisited by health extension workers every 3 months (Table 4).

**Table 4:** A multilevel logistic regression analysis of childhood diarrhea in high and low hotspot districts of Amhara Region, northwest Ethiopia, February–March 2015

Variables	Model 2	Model 3	Model 4
	AOR (95 % CI)	AOR (95 % CI)	AOR (95 % CI)
Individual-level factors			
Child factors			
Child’s age (months)			
0–5	0.28 (0.03–2.52)		0.55 (0.06–5.4)
6–11	3.94 (2.13–7.28)		5.87 (3.00–11.46)***
12–23	3.36 (2.12–5.30)		3.31 (2.07–5.29)***
24–35	1.75 (1.10–2.80)		1.80 (1.11–2.92)*
36–47	1.32 (0.81–2.15)		1.44 (0.87–2.38)
48–59	1.00		1.00
Complementary feeding at			
≥6 months	1.00		1.00
<6 months	8.72 (8.96–12.76)		6.81 (4.52–10.25)***
Maternal/household factors			
Hand washing practice of six critical times			
Practicing at least three critical times	1.00		1.00
Fail to practice at least three critical times	1.68 (1.20–2.35)		1.70 (1.20–2.40)*
Comprehensive knowledge on diarrhea			
Knowledgeable	1.00		1.00
Limited knowledge	1.47 (1.10–1.97)		1.45 (1.06–1.97)*
Wealth index			
Rich	1.00		1.00
Middle	1.19 (0.84–1.69)		1.20 (0.84–1.72)
Poor	2.05 (1.41–2.97)		1.63 (1.12–2.36)*
Frequency of visiting of health extension workers			
Once in every 3 months	1.00		1.00
Once in every 6 months	1.25 (0.86–1.84)		1.43 (0.96–2.13)
Once above 6 months	1.94 (1.33–2.84)		1.71 (1.14–2.60)**
Community-level factors			
Latrine facility availability			
Improved latrine		1.00	1.00
Unimproved latrine		2.74 (1.94–3.87)	1.84 (1.23–2.75)**
Had no latrine facility		3.19 (2.24–4.54)	2.43 (1.61–3.63)***
Drinking water source			
Improved source		1.00	1.00
Unimproved source		1.88 (1.44–2.45)	1.73 (1.27–2.34)**
Measles vaccine status			
Vaccinated		1.00	1.00
Not vaccinated		1.97 (1.39–2.81)	3.81 (1.91–7.58)***
Rotavirus vaccine status			
Vaccinated		1.00	1.00
Not vaccinated		4.19 (3.17–5.55)	4.88 (3.56–6.68)***

Model 1 = empty model (without explanatory variable)

*p < 0.05; **p < 0.01; ***p < 0.001

The odds of having diarrhea in children living in households with unimproved water source were 1.73 times (AOR 1.73; 95 % CI 1.27–2.34) higher than in children living in households with improved water source. The odds of having diarrhea in children living in households with no latrine facility and unimproved latrine were 2.43 (AOR 2.43; 95 % CI 1.61–3.63) and 1.84 (AOR 1.84; 95 % CI 1.23–2.75) times higher, respectively, than in children living in households with improved latrine facility. Those children who did not receive measles vaccine were 3.81 times (AOR 3.81; 95 % CI 1.91–7.58) more likely to develop diarrhea than those children who received measles vaccine, and those children who did not receive rotavirus vaccine were 4.88 times (AOR 4.88; 95 % CI 3.56–6.68) more likely to develop diarrhea than those children who received rotavirus vaccine (Table 4).

In a multilevel analysis, the empty model (the null model) revealed that childhood diarrhea was not random across the communities (*τ*^2^ = 0.139, *p* < 0.001). Intra-cluster correlation based on estimated intercept component variance showed that the odds of childhood diarrhea could be attributed to the community-level factors. The full model, after adjusting for individual- and community-level factors, has shown that the variation in childhood diarrhea across the community remained statistically significant. The variance of both the individual- and the community-level random effect was 0.085 and the intra-cluster correlation coefficient was 2.3 %. About 35.1 % in the odds of childhood diarrhea variation across the communities was observed by the full model. Moreover, MOR also confirmed that childhood diarrhea was attributed to community-level factors. The MOR for childhood diarrhea was 1.5 in the empty model; this indicated that there is variation across communities compared to no variation across communities if MOR is 1. The unexplained community variation in childhood diarrhea decreased to an MOR of 1.4 when all factors were added to the null model (empty model). This value confirmed that there were also unexplained variations between communities that contribute for the occurrences of childhood diarrhea (Table 5).

**Table 5:** Results from random intercept model (measure of variation) for childhood diarrhea at cluster level by multilevel logistic regression analysis

Measure of variation	Model 1^a^	*p* value	Model 2^b^	*p* value	Model 3^c^	*p* value	Model 4^d^	*p* value
Community level								
Variance (SE)	0.139 (0.078)	<0.001	0.105 (0.070)	<0.01	0.053 (0.044)	<0.05	0.088 (0.071)	<0.05
Explained variation (PCV)	Reference		19.8		61.9		35.1	
ICC (%)	3.71		2.83		1.45		2.3	
MOR	1.54		1.47		1.31		1.40	
Model fit statistics								
DIC (-2log likelihood)	1963		1438		1733		1313	

*SE* standard error, *ICC* intra-cluster correlation, *MOR* median odds ratio, *DIC* deviation information criterion

^a^Model 1 is the empty model, a baseline model without any determinant variable

^b^Model 2 is adjusted for individual-level factors

^c^Model 3 is adjusted for community-level factors

^d^Model 4 is final model adjusted for both individual- and community-level factors

## Discussion

The overall prevalence of childhood diarrhea in our study area was 13.5 % (95 % CI 12.2–14.8 %), which is similar to the national prevalence reported by Demographic Health Survey result done in 2011 [7]. However, it is lower than studies conducted in different parts of Ethiopia, such as in Keffa Sheka zone of southern Ethiopia (15 %) [16], in Mecha District of northwest Ethiopia (18 %) [11], in Kersa District of western Ethiopia (22.5 %) [14], and in Benishangul Gumuz Regional State, northwest Ethiopia (22.1 %) [15]. The difference might be attributed to the difference in the socio-demographic characteristics and basic environmental infrastructure of study households, behaviors of care givers, and the study period. The lower prevalence may be due to the combination of interventions to improve child health, such as water, sanitation and hygiene interventions, breastfeeding, vitamin A supplementation, and vaccines for diarrhea (rotavirus vaccine) [44]. For instance, the rotavirus vaccine was launched in Ethiopia at the beginning of 2014 [33] and sanitation facility coverage improved from 2 % in 2000 to 46 % in 2012 [31] in the past decade in Amhara Region.

This study revealed that the prevalence of childhood diarrhea did not show a statistically significant variation between high (14.3 %) and low (12.3 %) hotspot districts. The possible explanation may be due to the government strategies to improve child health, including prevention of childhood diarrhea, through the implementation of health extension packages that could produce a similar impact in the study area and possibly in the region and the country [32]. The other possible reason may be due to the inherent drawback of cross-sectional study design used in this study that reflects one time or snapshot results unlike spatial analysis that used longitudinal retrospective data of 7 years [45].

This study confirmed that the variation in childhood diarrhea is attributed to individual- and community-level factors. In the full model, both individual- and community-level factors accounted for about 35 % of the variations observed for childhood diarrhea. Child age was one of the factors associated with childhood diarrhea in this study. The risk was most at age segments of 6–11 months, high at 12–23 months and 24–35 months, and least at 0–5 months compared to children aged above 47 months. This finding is in agreement with other studies [8, 14, 46–51]. The low risk of diarrhea during the age 0–5 months may be attributed to the protective effect of exclusive breastfeeding and less exposure of children to contaminated agents. The peak risk of diarrhea among children between the ages of 6–11 months could be due to crawling on the ground or walking which increases the probability of getting and contacting with filth materials that may expose to pathogenic microorganisms [52]. In addition, the complementary food practices which usually start after 6 months may increase the incidence of diarrhea diseases if the food preparation could not be done in a hygienic manner. In support of this, there exists evidence that the risk of complementary feeding as a result of contamination increases diarrhea in this age group [47, 53–55] and decreases subsequently after 6–11 months; this is probably because the children begin to develop immunity to pathogens after repeated exposure [55].

The findings of this study suggest that the risk of diarrhea among children who started complementary foods before 6 months was higher than those who started at or after 6 months. This finding is similar to other studies [8, 11, 46]. The time to initiate complementary feeding practice can reflect the duration of exclusive breastfeeding, which is an effective means of protecting children from diarrheal disease [56]. There is evidence of the protective effect of exclusive breastfeeding from diarrhea in the presence of improved water supply and sanitation; this effect was found statistically significant compared to the partially breastfed and the non-exclusive breastfeeding children [57, 58]. The possible justifications could be due to the fact that unhygienic complementary feeding practices increase the risk of diarrhea [59], exclusive breastfeeding even later than 6 months can reduce exposure to infection in areas where environmental sanitation is very poor, and the immunologic properties of breast milk protect children against infection.

The results of this study showed that the risk of diarrhea among children from poor households was higher than children from rich households, and this is in agreement with other studies [8, 60, 61]. This may indicate that people in rich households are more likely to apply better hygienic practices and environmental sanitation because of their living standard, and this may prevent childhood diarrhea occurrence [62, 63].

The finding of the study is consistent with earlier studies, which found higher odds of childhood diarrhea among children whose mothers failed to wash their hands at hand washing critical times [60, 64–66]. Moreover, this study found that the risk of diarrhea among children whose mothers/caretakers had less knowledge on prevention methods of diarrhea was higher compared to children whose mothers/caretakers had knowledge on prevention methods of diarrhea. Finally, this finding is similar to other studies, which found that respondents with less knowledge on predisposing factors of diarrhea were more likely to have poor hand washing practice [50, 67]

Ethiopia has been implementing a community-level health intervention package (referred to as “Health Extension Program”) to improve the health of children in particular by deploying health extension workers. Of the 16 packages, seven involve hygiene and environmental sanitation (excreta, solid and liquid waste disposal, water supply, food hygiene, housing, personal hygiene, vector and rodent control) [32]. Risk of diarrhea in children was linked to the frequency of visits by health extension workers. For instance, households visited every 3 months were able to decrease diarrhea by 41 % compared to households visited one time in 6 months in this study. The frequency of visitation by health extension workers might have increased the implementation of health extension packages by households in the domain. There is evidence that childhood diarrhea reduced significantly among families in households that fully implemented basic health packages [13, 68].

The health risks from inadequate water, sanitation, and hygiene have been documented previously [69]. There is also evidence that shows the risk reduction of diarrheal disease being associated with improvements in both water and sanitation [70]. Environmental fecal-oral pathogen load varied at different scenarios: pathogen load was low in areas having greater than 98 % of improved water supply and sanitation coverage, but high in areas with incomplete coverage of either improved water supply or sanitation and very high in areas practicing open defecation [71]. The provision of improved water supply was found to reduce the risk of childhood diarrhea by 40 % in this study, which is similar to other findings that stated that provision of improved water supply was found to be effective in diarrhea disease reduction, particularly the provision of piped water supply [51, 60].

Households with unimproved latrine and absence of latrine at the community level were found to increase childhood diarrhea by 49 and 75 %, respectively, compared to households with improved latrines in this study. A similar study indicated the risk of exposure to high level of pathogens from fecal material in households with improved sanitation from neighbors who have no improved sanitation [60]. A meta-analysis done in 2014 showed that the overall effect for access to an improved sanitation facility on reduction in diarrhea morbidity was 28 % (RR 0.72, 95 % CI 0.59–0.88) [70].

Community immunity protects unvaccinated children and adults by reducing the spread of transmission and has also been noted after routine rotavirus immunization [27, 72] and measles immunization in several countries. The study found that the risk of diarrhea among children who did not receive measles vaccine was nearly four times higher compared to children who received measles vaccine. This finding is in agreement with other studies [73–75], which found that the incidence of diarrhea was high during outbreak of measles.

Studies showed rotavirus as the most common cause of vaccine-preventable diarrheal disease during dry seasons [1, 23]. Childhood diarrhea among children who received rotavirus vaccine was also less compared to children who did not receive rotavirus vaccine. This finding is in line with other findings [23–29], which found that diarrhea episodes among children were reduced after implementation of this vaccine during dry season. This study was carried out during dry season from February 2015 to April 2015. Since 2014, Ethiopia has become one of the 49 eligible countries for funding by the Global Alliance for Vaccines and Immunization (GAVI) to begin universal rotavirus immunization [33].

Analyzing individual- and communal-level factors using a multilevel model which is very important to control unexplained variations of the higher level for preventing misleading associations is the strength of this study. One of the limitations of this study is the cross-sectional nature of the study, and it shares the drawbacks of similar cross-sectional studies. Underestimation of childhood diarrhea prevalence might be another limitation of this study since data were collected in the dry season (February to March). Comprehensive knowledge of diarrhea which include causes of diarrhea, means of transmission and methods of preventions of diarrhea, and hand washing practices used in the analysis were self-reported by the respondent; self-reported data have been found to introduce inaccuracy and bias into estimates of behavior [76].

## Conclusions

The prevalence of childhood diarrhea remains high in the study area. The prevalence of childhood diarrhea did not show a statistically significant variation between high and low hotspot districts. Both individual- and communal-level factors play a significant role in the occurrence of childhood diarrhea, which accounted for 35 % variation across the communities. Age of child between 6 to 35 months, complementary feeding initiation below 6 months, inadequate hand washing practices, less knowledgeable mothers on prevention methods of diarrhea, poorest wealth status of households, and household revisit time above 6 months by health extension workers were the factors that increased the odds of childhood diarrhea at individual level, whereas lack of improved water supply and improved sanitation facilities and unvaccinated children with measles and rotavirus vaccine were the factors associated with childhood diarrhea at the community level.

Therefore, the reduction of childhood diarrhea requires combination of interventions at individual and community level: education of mothers on proper time of complementary feeding, causes of diarrhea, means of transmission and prevention methods of diarrhea, improved economic status of households, provision of improved drinking water supply and improved sanitation, increasing the coverage of measles and rotavirus vaccine.

## Additional files

Additional file 1: Additional file on results of high and low hotspot districts in the study area. (XLS 31 kb) Additional file 2: Questionnaire (English_version) (DOC 238 kb)

## Abbreviations

AOR: adjusted odds ratio
CI: confidence interval
GAVI: Global Alliance for Vaccines and Immunization
ICC: intra-cluster correlation
MOR: median odds ratio
PCV: proportional change in variance
WHO: World Health Organization
