# Supplementary material for: Childhood diarrhea in high and low hotspot districts of Amhara Region, northwest Ethiopia: a multilevel modeling
Source: J Health Popul Nutr. 2016 May 16;35:13. doi: 10.1186/s41043-016-0052-2 (PMC5025988; doi:10.1186/s41043-016-0052-2)
Supplement: Additional file 2: — Questionnaire (English_version) (DOC 238 kb) [file 41043_2016_52_MOESM2_ESM.doc]

### B. English Version Questionnaire:

### For objective II

Structured Questionnaire to identify Factors to childhood Diarrhea

**Addis Ababa University**

**Ethiopian Institute of Water Resources**

#### Subject Information Sheet

Good morning/Good afternoon. My name is__________________ and I am a member of a team conducting research to assess the determinants of diarrhea among under-five children. The study is conducted one of the PhD student in collaboration with Addis Ababa University, Ethiopian Institute of Water Resources.

The purpose of my visit today is to take information from you on health (diarrhea) of children less than five years of age. If you are willing to participate, I will ask you few questions. I will visit your home and backyard environment to collect information. In the study if you are found to have a certain health problems, appropriate educational counselling and education will be given to you. However, no financial payment will be made for your participation.

Your name will not be written on this form and will never be used with any information you may tell me. You do not have to answer any questions that you do not want to answer and you may end this interview at any time you want. However, your honest answer to these questions and your continuous interest to participate in study will help as for better understanding of the determinants of diarrhea eventually help in designing and implementing appropriate intervention programme to alleviate the problem.

We would very much appreciate your participation in this research by genuinely responding to the interviews. Your participation in the study is fully based on your interest and choice. It would take 20-30 minutes to complete the questionnaire. If you have any question during my interview and observation of the home and backyard of the environment, you can ask me at any time so that I can elaborate it. It is also possible to communicate the principal investigator through Tel +251911 364097

**Questionnaire ID………………………….**

#### Informed Consent Form

With the due understanding of the aforementioned information, would you be willing to participate in the study?

Yes

**Signature/Finger print of the participant**

Signature/Finger print ____________________Date ___________________

(Proceed to the interview)

No (Terminate the interview)

**Signature of the interviewer**

Name ______________________Signature ________________ Date __________

**Supervisors/Researcher remark and signature**

------------------------------------------------------------------------------------------------------------------------------------------------------------------------------------------------------------------------------

Name ______________________ Signature _______________ Date __________

| 001. | Questionnaire Number | …………………………. |
| --- | --- | --- |
| 002. | Type | 1. case 2. control |
| 003 | Residence address | 1. Rural ( Village ………………House No…….) 2. Urban (Village ………………House No…….) |

Time started………………………Time ended………………………………

Instructions: Put the respondents answer inside the box

#### Part I: Socio-demographic and economic Variables

| S. No | Variables | Answer | Response Options | Code | Skip |
| --- | --- | --- | --- | --- | --- |
| Q101 | Relation of the respondent to the child |  | 1. Mother 2. Caregiver | S101 |  |
| Q102 | Mother’s/caregivers Age |  | …………………………. | S102 |  |
| Q103 | Marital status of mothers/caregiver |  | 1. Married 2. Single  3. Widowed 4. Separate  5. Divorced | S103 |  |
| Q104 | Religion of the parents/caregiver |  | 1. Orthodox 2. Protestant  3. Muslim 4. Catholic  9. Others (specify) ____________ | S104 |  |
| Q105 | Age of the child’s father |  | …………………………………… | S105 |  |
| Q106 | Ethnic group of the parents/caretakers |  | 1. Amhara 2. Agew  3. Oromo 4. Tigrie  9. Others (specify) __________ | S106 |  |
| Q107 | Total family size of the HHs |  | Write in number _________ | S107 |  |
| Q108 | Total number of < 5 children |  | Write in number _________ | S108 |  |
| Q109 | Educational level of mother /caregiver |  | 1. unable to read and write 2. able to write and read 3. Primary (1-8 grade) 4. secondary (9-10) 5. Higher education (diploma and above) | S109 |  |
| Q110 | if you have a husband, Educational level of your husband |  | 1. unable to read and write 2. able to write and read 3. Primary (1-8 grade) 4. secondary (9-10) 5. Higher education (diploma and above) | S110 |  |
| Q111 | Maternal/caregiver occupational status |  | 1=not working, 2= daily labourer 3= farmer, 4 =employee/professional  9. Other (specify)_______ | S111 |  |
| Q112 | If you have a husband, occupational status of your husband |  | 1=not working, 2= daily labourer/manual, 3= farmer, 4 =employee/professional, 5. Merchant, 9. Other (specify)______ | S112 |  |
| Q113 | Wealth status of the house  Do you have following kinds of livestock? If yes, state size of herd. |  | Presence of own farmland in (hec)………..  Own toilet facility (yes=1/no=2)  Bank account (yes=1/no=2)  Mobile phone(yes=1/no=2)  Electricity(yes=1/no=2)  Roof of house with corrugated iron sheet (yes=1/no=2)  Number of cows/oxen…………….  Number of horses/ mules/donkeys………  Number of goats/sheep ………………  Number of chicken…………… | S113a  S113b  S113c  S113d  S113e  S113f  S113g  S113h  S113i  S113j |  |
| Q114 | Monthly income of the household |  | ………………………………. | S114 |  |
| Q115 | Does the family have a radio? |  | 1. Yes 2. No | S115 |  |
| Q116 | Does the family have TV? |  | 1. Yes 2. No | S116 |  |

#### Part II. Information of the index child

Index Child: Ask the mother/caregiver about the child with diarrhea, or if there is no child with diarrhea, ask about the child who is younger than others.

Diarrhea- is defined as having three or more loose or watery stools in a twenty-four hours period, as reported by the mother/caregiver of the child

| S. No | Variables | Answer | Response Options | Code | Skip |
| --- | --- | --- | --- | --- | --- |
| Q201 | Age of index child (months) |  | …………………………. | C201 |  |
| Q202 | Sex of index child |  | 1. Male 2. Female | C202 |  |
| Q203 | Did your child receive measles vaccine? |  | 1. Yes 2. No | C203 |  |
| Q204 | Did your child receive Rota virus vaccine? |  | 1. Yes 2. No | C204 |  |
| Q205 | Do you (the mother/caregiver) have a history of diarrhea in the past two weeks? |  | 1. Yes 2. NO | C205 |  |
| Q206 | Is there any family member who has a history of diarrhea in the past two weeks? |  | 1. yes 2. no | C206 |  |
| Q207 | Does your child have/had diarrhea? |  | 1. Yes 2. No | C207 |  |
| Q208 | If yes Q207, how many times a day he/she passes stool? |  | 1. Three times  2. More than three times  9. Don't know | C208 |  |
| Q209 | If yes Q207, For how long the diarrhea last? |  | 1. Less than 14 days 2. Greater than 14 days | C209 |  |
| Q210 | The type of diarrhea that the child had |  | 1. Watery  2. Blood and mucus  9. Others (specify)...... | C210 |  |
| Q211 | What actions do you take to treat/stop the diarrhea? |  | 1. Take him/her to health institution  2. Take him/her to traditional healer  3. Increase feeding  4. Give him/her ORS  5. Give him/her cereal based fluids  6. Stop/decrease feeding  7. Homemade treatment  9. Other (specify)………….. | C211 |  |
| Q212 | Have you heard about ORS? |  | 1. Yes 2. No | C212 |  |
| Q213 | If yes Q212, have you ever used it? |  | 1. Yes 2. No | C213 |  |
| Q214 | Have you heard about Zinc? |  | 1. Yes 2. No | C214 |  |
| Q215 | If yes Q214, have you ever used it? |  | 1. Yes 2. No | C215 |  |

#### Part III. Behaviors and practices of mothers or caregivers

| S.No | Questions (Variables) | Answer | Response options | CODE | Remark |
| --- | --- | --- | --- | --- | --- |
| Q301 | Does the child take other food than breast milk? |  | 1. Yes 2. No (If No skip to Q306) | M301 |  |
| Q302 | When did the child start other foods? |  | 1. Before six months 2. After six months | M302 |  |
| Q303 | Do you prepare food separately for the child, using a separate material? |  | 1. Yes 2. No | M303 |  |
| Q304 | What food/fluid is the child mostly receiving? |  | 1. Cow's milk(1 =yes 2= no)  2. Powder milk (1 =yes 2= no)  3. Adults' food (1 =yes 2= no)  4. Gruel (1 =yes 2= no)  9. Other (specify)……………… | M304a M304b M304c M304d M304e |  |
| Q305 | What do you use to feed the child? |  | 1. Hand  2. spoon  3. Cup  4. Bottle  9.Other (specify)................................ | M305 |  |
| Q306 | Mainly, when do you wash your hands? (More than one answer is possible) |  | 1. Before handling of food (Yes/No) 2. Before serving food(Yes/No) 3. Before feeding a child (Yes/No) 4. After visiting the latrine(Yes/No) 5. After eating (Yes/No) 6. After cleaning child feces(Yes/No)   9. Other(Specify) ………………… | M306a  M306b  M306c  M306d  M306e  M306f  M306g |  |
| Q307 | How often do you wash your hands   1. Before handling of food? 2. Before serving food 3. Before fetching a water 4. After visiting the latrine 5. After eating 6. After cleaning child feces |  | 1. 1. Always 2. 2. Sometimes 3. 3. Never | M307a  M307b  M307c  M307d  M307e  M307f |  |
| Q308 | What do you usually use to wash your hands |  | 1. usually water only 2. usually soap and water 3. sometimes soap and water 4. Other (specify)…………………… | M308 |  |
| Q309 | Do you get counseling from health workers about the advantages of washing hands by means of soap or ash? |  | 1. Yes  2. No  3. Not known/not sure | M309 |  |
| Q310 | Do you know that flies can transmit diseases? |  | 1. Yes 2. No | M310 |  |
| Q311 | If “Yes”for Q310, can you tell me the name of the diseases? |  | 1. Diarrhea (yes=1, No=2)  2. Typhoid fever(yes=1, No=2)  3. Cholera(yes=1, No=2)  4. Trachoma(yes=1, No=2)  5. Do not know the names,  9.Other (specify)………………….. | M311a  M311b  M311c  M311d  M311e  M311f |  |
| Q312 | Do you know that excreta of children can be a cause of diseases? |  | 1. Yes 2. No | M312 |  |
| Q313 | If the answer of Q312 yes, can you tell me the name of these diseases? |  | 1. Diarrhea (yes=1, No=2)  2. Typhoid fever(yes=1, No=2)  3. Cholera(yes=1, No=2)  4. Trachoma(yes=1, No=2)  5. Do not know the names,  9. Other (specify)…………………… | M313a  M313b M313c M313d M313e M313f |  |
| Q314 | If the answer of Q312, no  Do you know diarrhea? |  | 1. Yes 2. No | M314 |  |
| Q315 | If yes for Q314, what are the main signs/symptoms of diarrhea? (multiple responses are possible) |  | 1. Three or more unformed stools within a day (yes=1, No=2) 2. Vomiting(yes=1, No=2) 3. Abdominal pain (yes=1, No=2) 4. Fever(yes=1, No=2) 5. Cramps(yes=1, No=2) 6. Blood in stool (yes=1, No=2) 7. Nausea (yes=1, No=2) 8. Other (specify )………………….. | M315a  M315b  M315c  M315d  M315e  M315f  M315g  M315h |  |
| Q316 | What do you think causes diarrhea in young children? |  | Indigestible foods (yes=1, No=2)  Teething(yes=1, No=2)  Germ infection(yes=1, No=2)  Worm infection (yes=1, No=2)  Other (Specify)……………… | M316a  M316b  M316c  M316d  M316e |  |
| Q317 | What do you think spreads diarrhea? |  | 1. contaminated water(yes=1, No=2) 2. contaminated food(yes=1, No=2) 3. contaminated environment (yes=1, No=2) 4. through flies (yes=1, No=2) 5. other (Specify)……………………. | M317a  M317b  M317c  M317d  M317e |  |
| Q318 | Do you think diarrhea is a hazard to the child’s health? |  | 1. Yes 2. No | M318 |  |
| Q319 | Do you know some of the ways for preventing diarrhea? |  | 1. Yes 2. No | M319 |  |
| Q320 | If yes Q319, can you tell me the prevention methods? |  | 1. personal hygiene 2. environmental and house hygiene 3. keeping the latrine clean 4. keeping utensils clean 5. covering of cooked foods | M320a  M320bM320cM320d  M320e  M320f M320g |  |
| Q321 | Do you think no cleaning of latrine can facilitate to spread diarrhea |  | 1. Yes 2. No 3. Don’t know | M321 |  |
| Q322 | Do you think that washing hands after visiting latrine can prevent diarrhea? |  | 1. yes 2. no 3. don’t know | M322 |  |
| Q323 | Do you think that improper latrine utilization can transmit diarrhea? |  | 1. yes 2. no 3. don’t know | M323 |  |

#### Part IIV: Characteristics of Home and Environmental Health Conditions

| **S. No** | **Questions** |  | **Possible Answers** | **Code** | **Skip** |
| --- | --- | --- | --- | --- | --- |
| Q401 | Type of roof of the house (observation) |  | 1. Corrugated Iron Sheet  2. Tukul/thatched  9. Other(Specify) ………………….. | E401 |  |
| Q402 | Type of the wall (observation) |  | 1. Timber and mud 2. Timber and bamboo  3. Stone and mud 4. Stone and cement  5. Blockets 6. Bricks | E402 |  |
| Q403 | Type of house floor (observation) |  | 1. Earth /Soil 2. Cement/Brick  3. Wooden/Bamboo 4. Ceramic  9. Other(Specify) …………………….. | E403 |  |
| Q404 | How many rooms for use by the member of your household? (Excluding kitchen and store) |  | ________in number | E404 |  |
| Q405 | Do you have separate room which is used as Kitchen? |  | 1. Yes  2. No | E405 |  |
| Q406 | If you have livestock, where do they live? |  | 1. in the same room with family 2. there is separate room for them sleeves 3. outside the room/’beret laye’ | E406 |  |
| Q407 | What is your main source of drinking water? |  | 1. Piped in to dwelling  2. Public tap  3. Other Improved water supply (Protected spring & well)  4. unimproved (Unprotected spring & well, river/stream, Dam) | E407 |  |
| Q408 | How much time is required to fetch water from the house to the water source? |  | 1. Piped in to dwelling 2. 1-15 minutes 3. 16-30 minutes 4. ½-1hour below 5. 1 hour and above | E408 |  |
| Q409 | Average household water use per day per person for drinking, cooking and personal hygiene |  | In liters………………… | E409 |  |
| Q410 | How could you draw water from the storage? |  | 1. Pouring 2. Deeping  9. Others (specify) ………………….. | E410 |  |
| Q411 | If the of Q410 is deeping , do you use separate cap for drawing water from water storage container? |  | 1. yes 2. no | E411 |  |
| Q412 | Do you treat water in any way to make it safer at home? |  | 1. Yes 2. No | E412 |  |
| Q413 | If the answer for Q# 413 is yes, what activities do you practiced? |  | 1. Boiling  2.Chemical treatments  3. Filtering using cloth  4. filtering using sand, gravel or ceramics  5. Sun radiation treatment  6. Sedimentation  9. Others (specify) …………………….. | E413 |  |
| Q414 | Does the drinking-water storage container have a cover? |  | 1. Yes 2. No | E414 |  |
| Q415 | How is the cleanness of water storage container (inside and outside? (observation) |  | 1. very clean 2. presence of some filth material 3. it is dirty | E415 |  |
| Q416 | Does water storage container put above 40 cm on floor? |  | 1. Yes 2. No | E416 |  |
| Q417 | How frequently clean storage container material? |  | 1. always before fetching water 2. daily 3. weekly 4. if the material looks dirty | E417 |  |
| Q418 | Do you wash your hands before fetching water? |  | 1. yes 2. no 3. never | E418 |  |
| Q419 | Do you have latrine? (observation) |  | 1. Yes 2. No | E419 |  |
| Q420 | If the answer for Q# 419 is yes, type of latrine the households use? (Observation) |  | 1.Private traditional pit latrine  2. Private wooden slab latrine  3. Private cement slab latrine  4.Private VIP latrine  5. Shared wooden slab latrine  6. Shared VIP latrine  7. Flash to sewerage system  9. Other (Specify)……………….. | E420 |  |
| Q421 | If yes Q419, is it in use? (observational)  *feces is not seen around the house (or in the compound) and feces in the pit of the latrine |  | 1. Yes in use 2. Not in use | E421 |  |
| Q422 | How often is the latrine cleaned? |  | 1. Every day 3. 1-2 time per month 2. 1-2 times per week 4. Not cleaned | E422 |  |
| Q423 | If yes Q418, how is the cleanliness/ hygienic condition of the latrine? (observational) |  | 1. Clean 2. Not clean | E423 |  |
| Q424 | If the answer for Q# 419 is no, where do the households defecate? |  | 1. Designated area 2. Bush  3. At the back yard 4. Open field  9. Others (specify)_________ | E424 |  |
| Q425 | Are your children able to use the latrine on their own? |  | 1. yes 2. no | E425 |  |
| Q426 | If yes for Q425, how do you care after using latrine? |  | 1. with water 2. with soft tissue/paper 3. Not clean at all 4. other, specify…………….. | E426 |  |
| Q427 | If no for Q425, where do they defecate? |  | 1. disposing into latrine 2. burring in pit 3. disposing into drainage 4. put into solid waste 5. dispose into housing compound | E427 |  |
| Q428 | Where do you dispose of waste water? |  | 1. Sewage system  2. Pond  3. Garden  9. Other: …..… | E428 |  |
| Q429 | Do you use stool as fertilizer |  | 1. Yes 2. No | E429 |  |
| Q430 | Distance between toilet and water source |  | 1. 0 – 5 meters 2. 6 – 10 meters  3. 11-20 meters 4. 21 - 29 meters  5. 30 meters or more | E430 |  |
| Q431 | Mainly, how do the household dispose solid wastes? |  | 1. In a private pit 2. Common pit  3. Composting 4. Buried  5. Burning  6. Open field disposal  7. By other organized agents  9. Other (specify) _____________ | E431 |  |
| Q432 | Is there hand washing facility near by a latrine?(observation) |  | 1. Yes 2. No | E432 |  |
| Q433 | If yes for Q 432, is there water in the container? (observation) |  | 1. Yes 2. No | E433 |  |
| Q434 | If yes for Q432, is there moisture observed in the ground? (observation) |  | 1. Yes 2. No | E434 |  |
| Q435 | If yes for Q432, at hand washing facility? |  | 1. is there a soap container? (1 =Yes 2=no) 2. is there a soap? (1 =Yes 2=no) | E435a  E435b |  |
| Q436 | Does food preparation utensil have their own rack? (observation) |  | 1. yes 2. no | E436 |  |
| Q437 | How is the cleanliness of the rack? (observation) |  | 1. clean 2. dirty materials are their | E437 |  |
| Q438 | How frequently health extension workers visit the household? |  | 1. once per month 2. once per three month 3. once per six month 4. once per year 5. once more than one year | E438 |  |
